# Dual phosphodiesterase type 5 inhibitor therapy for refractory pulmonary arterial hypertension: a pilot study

**DOI:** 10.1186/s12890-015-0037-8

**Published:** 2015-05-14

**Authors:** Mai Kimura, Yuichi Tamura, Makoto Takei, Tsunehisa Yamamoto, Tomohiko Ono, Jun Fujita, Masaharu Kataoka, Masataka Kuwana, Toru Satoh, Keiichi Fukuda

**Affiliations:** Department of Cardiology, Keio University School of Medicine, 35 Shinanomachi, Shinjuku-ku, Tokyo 160-8582 Japan; Department of Allergy and Rheumatology, Nippon Medical School, Tokyo, Japan; Department of Cardiology, Kyorin University School of Medicine, Tokyo, Japan

**Keywords:** Phosphodiesterase type 5 inhibitor, Pulmonary arterial hypertension, New treatment option

## Abstract

**Background:**

Recent vasodilating drugs have improved prognosis of Pulmonary arterial hypertension (PAH). Some reports describe the merits of combination therapies for PAH, and this study evaluated the efficacy and safety of phosphodiesterase type 5 inhibitors (PDE5i) combination therapy, using sildenafil and tadalafil, for multi-drug-resistant PAH.

**Methods:**

We retrospectively analyzed 7 consecutive refractory patients with PAH administered either sildenafil 60 mg or tadalafil 40 mg as well as both ERA and prostanoid as combination therapies. All were started on the dual PDE5i (sildenafil and tadalafil at maximum dose).

**Results:**

Treatment was generally well tolerated without severe adverse events. On completion of the study, the seven patients received right heart catheterization and the 6-minute walk test (6WMT) 9.6 ± 1.4 months after initiation of the dual PDE5i therapy, showing significant improvements in hemodynamic parameters and exercise tolerance. Mean pulmonary arterial pressure and pulmonary vascular resistance decreased from 47.9 ± 9.7 to 41.7 ± 9.2 mmHg (*P* = 0.004) and 9.3 ± 2.7 to 6.7 ± 2.9 mmHg (*P* = 0.018), respectively. Cardiac index and 6MWT also increased from 2.8 ± 0.9 to 3.1 ± 0.8 L/min/m^2^ (*P* = 0.026) and 353 ± 60 to 382 ± 62 m (*P* = 0.014), respectively.

**Conclusion:**

The findings support dual PDE5i therapy as a new treatment option for refractory PAH.

## Background

Pulmonary arterial hypertension (PAH) is a progressive and fatal disease characterized by degeneration of the pulmonary arteries and subsequent increased pulmonary vascular resistance (PVR). These adverse effects lead to an increase in pulmonary arterial pressure (PAP) and high ventricular pressure overload, resulting in irreversible right heart failure.

The pathogenesis of PAH is partially characterized by the reduced expression of nitric oxide synthase in the pulmonary arterial vascular endothelial cells [1], which leads to impaired release of nitric oxide in pulmonary arteries [2]. From these findings, phosphodiesterase type 5 inhibitors (PDE5i) were proposed as potentially potent drugs against PAH that would act by increasing the cyclic guanosine monophosphate (cGMP) levels to induce the vasodilating [3] effects of endogenous nitric oxide.

Controlled trials of sildenafil, tadalafil and valdenafil for the treatment of patients with PAH showed improvements in exercise capacity, hemodynamic parameters and clinical outcome [4-7]. However, the currently approved dose of sildenafil is derived from that used to improve exercise capacity in the clinical study, and current reports suggest that the 6-minute walk test (6MWT) is insufficient as a surrogate endpoint in clinical trials for PAH [8,9], instead recommending improvements in PVR and time to clinical worsening as endpoints. Indeed, the SUPER-1 [5] and SUPER-2 [10] trials showed dosage-dependent improvements in hemodynamics (both in PAP and PVR) with a dose fourfold higher than the currently approved clinical dose. Together, these results suggest that high-dose PDE5i could play a role in salvage therapy for multi-drug refractory PAH, realized by taking the PDE5 inhibitors, sildenafil and tadalafil, at the maximum approved dose.

This report describes the results of a retrospective study of the effects of dual PDE5 inhibitor therapy with sildenafil and tadalafil as a salvage therapy for multi-drug-resistant PAH.

## Methods

This study is a retrospective study. This study followed the ethical standards of the responsible committee on human experimentation (KEIO UNIVERSITY SCHOOL OF MEDICINE AN ETHICAL COMMITTEE, Tokyo, Japan; the approval code is 20100008) and the Helsinki Declaration of 1975, as revised in 2000. And written informed consent was obtained from all patients in the study. A total of 142 patients with pulmonary hypertension were treated at Keio University Hospital (Tokyo, Japan) from April 2009 to May 2013, and 104 of these were diagnosed with PAH, classified according to the Dana Point classification of PH [11]. Of the PAH patients, 26.9% (n = 28) received triple combination therapy with prostanoids, ERA, and PDE5i. Among them, seven consecutive patients with refractory PAH were treated with both PDE5i used in combination as a salvage therapy. The refractory PAH was defined as the patients suffering from the symptom of NYHA III or IV in spite of taking three kinds of PAH specific drugs. These seven patients had previously been treated with a single PDE5 inhibitor (PDE5i) at the maximum dose, either sildenafil (60 mg per day) or tadalafil (40 mg per day), as well as both endothelin receptor antagonist (ERA) and prostanoid as combination therapies. However, all patients retained a diagnosis of refractory PAH despite the three kinds of vasodilation therapies over at least 6 months. Accordingly, they were started on a salvage therapy of dual-administered PDE5i, sildenafil and tadalafil, used at the maximum dose, between August 2011 and December 2012. For our control patients group, we selected 10 patients with PAH (idiopathic or connective tissue disease associated) from the same cohort. The control patients were also performed continuous triple combination therapies and hemodynamic measurements but free from dual PDE5i therapy. And they were not performed any additional PH specific therapies during the observation period.

Right heart catheterization (RHC) and 6MWT were performed before and within several months after the initiation of combination therapy. Patient files and the clinical database were reviewed and data were collected on treatments for pulmonary hypertension (epoprostenol, prostanoids, ERA, PDE5i), adverse events, the New York Heart Association (NYHA) functional classification (FC), 6MWT, and hemodynamic data (mean PAP, PVR and cardiac index (CI)) assessed by RHC. Changes in hemodynamic data and 6MWT, before and after the initiation of combination therapy, were evaluated as endpoints. The NYHA FC of patients was allocated by the treating physician at the time of clinical assessment, according to the WHO Functional Classification of PAH (whereby an FC of 1–4 is derived from patient symptoms in relation to exercise capacity).

### Statistical methods

Statistical analyses were performed using SPSS version 21. Mean PAP, CI, and 6MWT exhibited normal distributions, so a paired *t*-test was performed to analyze these data. Only PVR data after the initiation of combination therapy did not exhibit a normal distribution, so the Wilcoxon signed-rank test was performed to analyze changes in PVR. A two-sided *P*-value of 0.05 was considered to be statistically significant.

## Results

A total of 142 patients with pulmonary hypertension were treated at our hospital from April 2009 to May 2013, and 104 of these were diagnosed with PAH. Of the PAH patients, 26.9% (n = 28) received triple combination therapy with prostanoids, ERA, and PDE5i. Among them, seven patients with refractory PAH were treated with both PDE5i used in combination as a salvage therapy. Table 1 details patient characteristics, including diagnosis, vasodilator treatment and treatment duration. The patients with refractory PAH were classified as idiopathic PAH (n = 3), heritable PAH (n = 1) or congenital systemic-to-pulmonary shunts (n = 1), associated with connective tissue disease (CTD-PAH; n = 1) or associated with neurofibromatosis (NF1-PH; n = 1). The mean patient age (± SD) was 39.9 ± 12.3 years (range 23–55 years), and all subjects were female. The mean disease duration (± SD) was 6.6 ± 2.6 years (range 3–10 years). One patient showed NYHA FC VI and all the others showed FC III. Six patients had taken sildenafil (60 mg per day), and one had received tadalafil (40 mg per day) previously. One patient had also received ambrisentan and the other six were given bosentan as ERA, while three patients received intravenous epoprostenol infusion and four had beraprost as prostanoids. The dosage of ERA and prostanoids were not changed either 3 months before the initiations of dual PDE5i therapy or during the study.

**Table 1:** **Baseline characteristics of the patients**

**Age (years)**	**Sex**	**PAH diagnosis**	**Disease duration (years)**	**NYHA**	**Hemodynamic parameters**			**6MWD (m)**	**Concomitant medication**	**Duration of PDE5i combination therapy (months)**
					**mPAP (mmHg)**	**PVR (WU)**	**CI (L/min/m** ^**2**^ **)**			
41	F	CTD-PAH	5	III	37	7.05	3.09	457	Sil, Be, Am	6
48	F	ASD-PAH	8	III	53	10.43	1.65	336	Sil, Be, Bos	12
38	F	NF1-PAH	9	III	55	13.8	2.29	330	Sil, Be, Bos	13
58	F	HPAH	10	IV	38	7.36	2.88	320	Sil, Epo, Bos	10
23	F	IPAH	3	III	40	7.3	2.94	390	Tad, Epo, Bos	14
26	F	IPAH	7	III	62	7.49	4.39	370	Sil, Epo, Bos	3
45	F	IPAH	4	III	50	12	2.22	267	Sil, Be, Bos	9

CTD: connective tissue disease, ASD: atrial septal defect, NF1: neurofibromatosis 1, HPAH: heritable pulmonary arterial hypertension, IPAH: idiopathic pulmonary arterial hypertension, mPAP: mean pulmonary arterial pressure, PVR: pulmonary vascular resistance, WU: Wood’s units, CI: cardiac index, 6MWD: six-minute walk distance, Sil: sildenafil, Tad: tadalafil, Be: beraprost, Epo: epoprostenol, Bos: bosentan, Am: ambrisentan.

Complications of therapy are shown in Table 2. No severe adverse effects were observed with the combination therapy of PDE5i including hypotension, but some patients suffered from headache and diarrhea during the observation period. There was no dropout due to death, lung transplantation, heart failure or drug escalation, and every patient could continue the therapy during the follow up period.

**Table 2:** **Complications of dual PDE5i therapy**

	**n**
**Headache**	3
**Diarrhea**	2
**Hot Flash**	4

**Figure 1:**
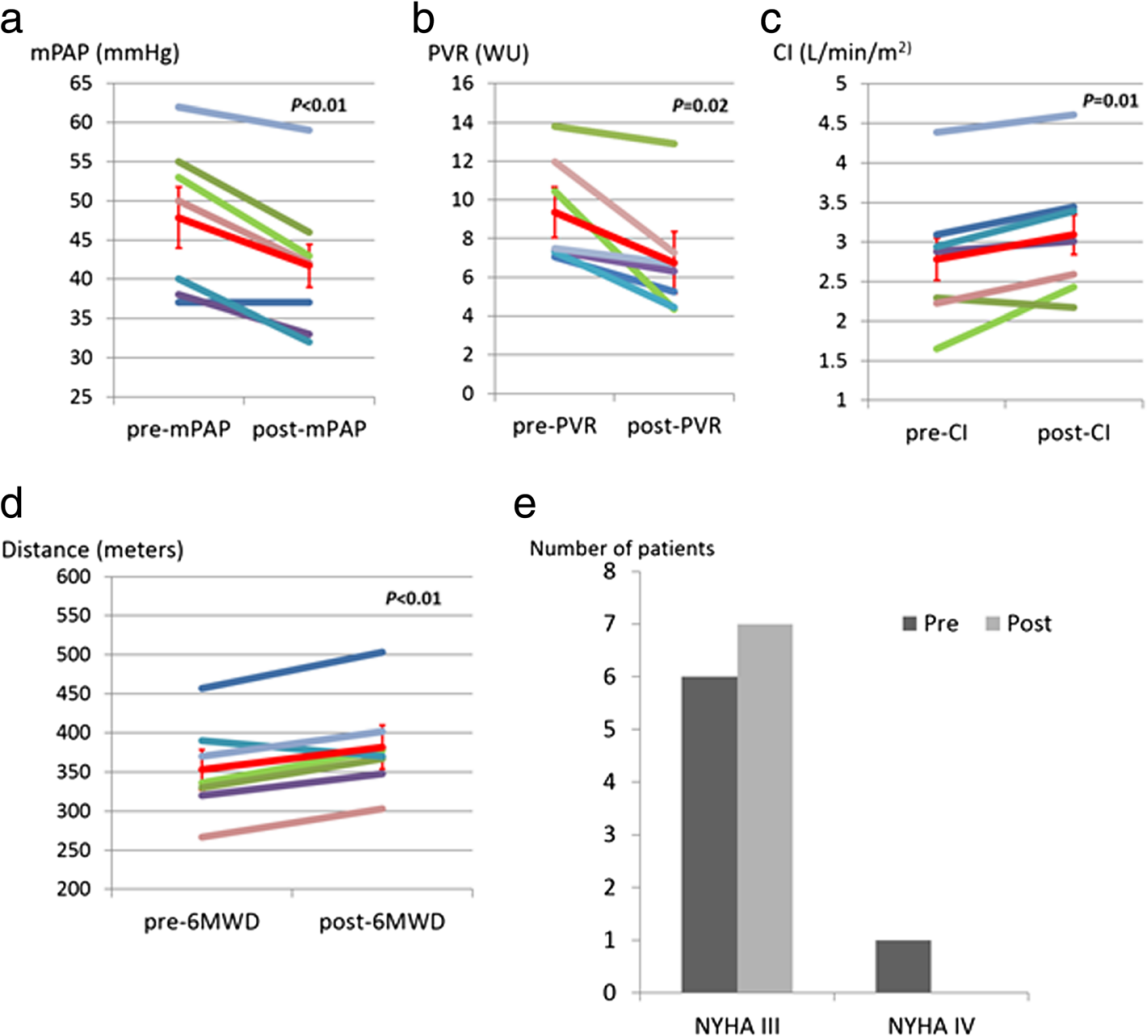
Hemodynamic and symptomatic changes before and after the dual PDE5i therapy. **a**. Effect of PDE5i dual therapy on mPAP. The red line indicates mean change of mPAP (± SD) from 47.9 ± 9.7 mmHg to 41.7 ± 9.2 mmHg. **b**. Effect of PDE5i dual therapy on PVR. The red line indicates mean change of PVR (± SD) from 9.3 ± 2.7 Wood’s Units to 6.7 ± 2.9 Wood’s Units. **c**. Effect of PDE5i dual therapy on CI. The red line indicates mean change of CI (± SD) from 2.8 ± 0.9 L/min/m^2^ to 3.1 ± 0.8 L/min/m^2^. **d**. Effect of PDE5i dual therapy on 6MWTD. The red line indicates mean change of 6MWD (± SD) from 353 ± 60 m to 382 ± 62 m. **e**. Effect of PDE5i dual therapy on NYHA functional class. mPAP: mean pulmonary artery pressure, PVR: pulmonary vascular resistance, WU: Wood’s units, CI: cardiac index, 6MWD: six minutes walk distance.

Follow up RHC and 6WMT were performed 9.6 ± 1.4 months after the initiation of the PDE5i combination therapy. The effect of combination therapy on mPAP, PVR, CI, and 6MWT are shown in Figure 1a to d, respectively, with all parameters showing significant improvement. Mean PAP improved from 47.9 ± 9.7 mmHg to 41.7 ± 9.2 mmHg, PVR from 9.3 ± 2.7 Wood’s Units to 6.7 ± 2.9 Wood’s Units, CI from 2.8 ± 0.9 L/min/m^2^ to 3.1 ± 0.8 L/min/m^2^ and 6MWT from 353 ± 60 m to 382 ± 62 m. The effect of combination therapy on NYHA FC was also shown in Figure 1e.

Finally we compared the patients with control patients who had similar characteristics but free from dual PDE5i therapy. Table 3 shows the details of patient characteristics, including treatment duration periods, baseline hemodynamic parameters and the changes after the contibuation of triple combination therapies. While the control patients also provided triple combination therapies with PDE5i, ERA and prostanoids, they acquired no hemodynamic improvements with the continuation of triple combination therapies. On the other hand, dual PDE5i therapy showed significant hemodynamic improvements compared with the control group.

**Table 3:** **Comparison between dual PDE5i therapy group and historical control**

	**Dual PDE5i group (N = 7)**	**Historical control (N = 10)**	**P value**
Age (years)	39.9 ± 12.3	33.0 ± 12.1	0.271
Number of Female	7 (100%)	10 (100%)	N/A
Follow-up period (months)	9.6 ± 4.0	10.7 ± 1.8	0.438
mPAP (mmHg)	47.9 ± 9.7	46.2 ± 7.8	0.700
ΔmPAP at follow-up (mmHg)	−6.1 ± 3.6	0.7 ± 3.3	0.001
PVR (WU)	9.3 ± 2.7	10.9 ± 5.0	0.458
ΔPVR at follow-up (WU)	−2.6 ± 2.1	1.5 ± 2.2	0.001
CI (L/min/m2)	2.8 ± 0.9	2.8 ± 1.0	0.983
ΔCI at follow-up (L/min/m2)	0.3 ± 0.3	−0.4 ± 0.6	0.007

mPAP: mean pulmonary artery pressure, PVR: pulmonary vascular resistance, WU: Wood’s units, CI: cardiac index.

## Discussions

In this uncontrolled, retrospective observational study of patients with PAH treated with both sildenafil and tadalafil, we achieved significant improvement in hemodynamic parameters as well as exercise tolerance despite the conditions of PAH being severe and refractory for combination therapy. In the previous SUPER [5] and PHIRST [7] studies, both sildenafil and tadalafil also improved 6MWD and FC in the short term. Nevertheless, half of the enrolled patients in PHIRST study group were initially treated with bosentan.

When to change treatment, or add an agent, in patients with PAH remain unanswered questions. However, the progressive and degenerative nature of PAH, as well as recent reports of improved therapeutic agents [12-15], provide encouragement to consider more aggressive approved therapy in patients showing no improvement. Combination therapies, with potentially additive or synergistic effects, are being explored. Indeed, targeted therapies significantly improved 6MWD and relative risk of death in a meta-analysis of studies in patients with PAH [16]. This is important considering that patients with poor exercise tolerance at baseline and whose 6MWD failed to improve after the initial treatments showed reduced long-term survival [10], suggesting a particularly poor prognosis without appropriate additional early therapy.

In this study, we found that additional treatment with a second PDE5i achieved significant improvement of PAH, suggesting that PDE5i dual combination therapy could work well as a salvage therapy in patients with severe and refractory PAH. One of the possible mechanisms underlying the observed improvement is that PDE5i are high-potency vasodilators, indicating that the approved dose of sildanfil (60 mg/day) or tadalafil (40 mg/day) does not fully inhibit PDE5 in the pulmonary vasculatures and the dual PDE5i are needed to sufficiently increase cGMP levels to have the desired therapeutic effect. In fact, the SUPER study [5] showed that sildenafil achieved dose-dependent improvements in hemodynamic parameters within a short time in a dose range of 60 mg to 240 mg/day. The PHIRST study [7] also showed statistically significant improvement in CI only in the tadalafil 40 mg group, but not for patients receiving 2.5, 10 or 20 mg/day. There are no data about the effectiveness of tadalafil used at more than 40 mg in a day. Such data strongly suggested that the effectiveness of PDE5 inhibition is dose dependent, and importantly, that PDE5i use would be more empowered at a higher dose than currently approved.

Another mechanism of the improvement of the improvement is the pharmacokinetic interactions between bosentan and sildenafil or tadalafil. Sildenafil is predominantly metabolized by cytochrome P450 (CYP) 3A4 and CYP2C9, while tadalafil is mainly metabolized by CYP 3A4. As bosentan induces both CYP3A4 and CYP2C9 [17], a pharmacokinetic interaction is possible between these agents. With the combination of bosentan, the maximum plasma concentration of sildenafil decreased by 55.4% [18] and the maximum plasma concentration of tadalafil also decreased by 26.6% [19], while combinations with bosentan and PDE5i were generally well tolerated. In this study, 86% of patients (n = 6) had already received bosentan before the initiation of PDE5i combination therapy, and 5 patients out of 6 received sildenafil and bosentan combination therapy. This fact suggested that compared with the PDE5i used alone, the effect of PDE5i used in combination was diminished by bosentan. In addition, changing from the single to dual PDE5i use recovered the degradation effect of PDE5 inhibition.

### Study limitations

The limitation of our study is this was a single center and retrospective study. Additional investigations such as a multi-centre placebo-controlled study should be performed to confirm the usefulness of PDE5i dual therapy. In addition, data from the STARTS-2 study [20] on sildenafil treatment for pediatric patients with PAH, showed a favorable prognostic improvement in patients given a medium dose for weight compared with the maximum dose group. Thus, the appropriate level of PDE5 inhibition needed to achieve the best prognosis remains unknown and could differ among patients groups. Further investigation is needed to determine the optimal dosing of PDE5i.

## Conclusion

In conclusion, this study demonstrates the safety and efficacy of combination therapy with sildenafil and tadalafil for the treatment of patients with severe PAH. The favorable efficacy-to-safety profile of the dual PDE5 inhibitors therapy provides a clinically meaningful new treatment option in addition to the currently approved treatment strategies.

## Abbreviations

PAH: Pulmonary arterial hypertension
PDE5i: Phosphodiesterase type 5 inhibitors
ERA: Endothelin receptor antagonist
6MWT: 6-minute walk test
PVR: Pulmonary vascular resistance
PAP: Pulmonary arterial pressure
RHC: Right heart catheterization
FC: Functional classification
CI: Cardiac index
NF1: Neurofibromatosis 1
CTD: Connective tissue disease
